# Concomitant Abdominoplasty and Repair of Midline Abdominal Wall Defects

**DOI:** 10.1007/s00266-026-06000-0

**Published:** 2026-06-30

**Authors:** Sandra Maria Tsoti, Ioanna Vasilaki, Andreas Foustanos

**Affiliations:** 1 National and Kapodistrian University of Athens (NKUA), Athens, Greece; 2 Plastic Surgery Department, Iaso Hospital, Athens, Greece

**Keywords:** Abdominoplasty, Diastasis recti, Midline hernia repair, Modified mayo technique, Mesh-free reconstruction, Long-term follow-up

## Abstract

**Background:**

Diastasis recti and midline abdominal wall hernias are frequently encountered in patients undergoing abdominoplasty, particularly postpartum women and individuals following significant weight loss. The associated musculoaponeurotic laxity results in functional abdominal wall weakness and contour deformities.

**Methods:**

We describe a surgical technique combining abdominoplasty with midline fascial repair, performed without mesh reinforcement. The procedure utilizes a modified overlapping Mayo double-suturing method to restore midline strength and contour. This paper presents a 39-year single-surgeon experience, including long-term clinical outcomes.

**Results:**

A total of 120 patients underwent the procedure between 1986 and 2021. All were discharged within 72 hours postoperatively and followed up regularly (range: 1–39 years). There were no recurrences of diastasis of rectus abdominis muscles (DRAM) or hernia and no cases of vascular compromise. Seromas occurred in 20% of cases and were successfully managed conservatively with drainage.

**Conclusion:**

This combined approach allows for durable anatomical and aesthetic correction of abdominal wall defects without prosthetic materials. The long-term outcomes presented support the safety, effectiveness, and reproducibility of the technique in appropriately selected patients.

**Level of Evidence IV:**

This journal requires that authors assign a level of evidence to each article. For a full description of these Evidence-Based Medicine ratings, please refer to the Table of Contents or the online Instructions to Authors www.springer.com/00266.

## Introduction

Abdominoplasty is a widely performed aesthetic procedure, increasingly sought after due to rising obesity rates, massive weight loss following bariatric surgery, and an aging population seeking body contour restoration. Multiple factors including significant weight fluctuations, pregnancies, aging, and prior abdominal surgeries can lead to excess, lax skin, visible striae, and weakening of the musculoaponeurotic system. A common coexisting finding is diastasis of the rectus abdominis muscles (DRAM), typically resulting from increased intra-abdominal pressure. DRAM is characterized by thinning and widening of the linea alba, often exceeding 2 cm, which leads to a central abdominal bulge. It is most prevalent in postpartum women and persists in up to 33% of cases [1]. DRAM is not merely a cosmetic concern. It may cause functional impairments such as core instability, weakened pelvic and lumbar support, and an increased risk of midline hernias [2]. Surgical correction of DRAM during abdominoplasty is therefore recommended to restore abdominal wall strength and contour [3]. This dual approach addresses both soft tissue laxity and underlying structural defects, contributing to improved function and aesthetic outcomes.

Fascial laxity and prior abdominal wall surgeries are significant risk factors for the development of abdominal wall hernias [4]. According to the European Hernia Society classification, these hernias are broadly divided into primary and incisional types and further stratified by anatomical location and size [5]. Incisional hernias occur in up to 20% of patients following abdominal surgery and demonstrate a female-to-male ratio of 3:1 [6–8]. When treated in the acute setting, hernia repairs are associated with higher morbidity and mortality than those performed electively [9]. Given the rising incidence of abdominal wall hernias, it is essential to utilize surgical techniques that preserve native anatomy and minimize recurrence. Over recent decades, hernia repair techniques have advanced significantly, particularly following the introduction of the Rives–Stoppa approach in the 1980s. Various modifications have since been described, including anterior and posterior component separation techniques, with or without the use of prosthetic mesh [10]. When a hernia is identified preoperatively or intraoperatively during abdominoplasty, the exposure allows for an ideal opportunity to repair the fascial defect directly, improving both the structural and aesthetic outcomes of the abdominal wall [11, 12]. This paper describes a surgical technique for abdominoplasty with concomitant repair of DRAM and abdominal wall hernias using local tissues, without prosthetic materials, while preserving vascular supply to the umbilical stalk and restoring abdominal wall integrity.

### Patient Data

A retrospective analysis was conducted on 120 patients who underwent abdominoplasty with concomitant repair of diastasis recti and/or midline abdominal wall hernias between 1986 and 2021 at Iaso Hospital in Athens. Of these, 54 patients were found to have incidental midline hernias identified intraoperatively. The cohort included 108 women (90%) and 12 men (10%), aged 35 to 61 years. Inclusion criteria consisted of postpartum women with large diastasis (Rectus Diastasis Classification W3, >5 cm) who had no future plans for pregnancy, and patients who had achieved a stable, normal-range BMI following significant weight loss [13]. Assessment of recurrence risk factors revealed that 55% of patients were smokers, three had undergone recent massive weight loss after bariatric surgery, and two had diabetes mellitus. Based on Chevrel’s classification, the incidental hernias were small to medium in size and located in the epigastric (M2) or umbilical (M3) region. The diastasis width ranged from 5 cm to 15 cm. All patients were evaluated preoperatively via thorough medical history, physical examination, and laboratory work-up. Standardized digital photographs were obtained to document preoperative appearance, intraoperative findings, and postoperative outcomes. All patients provided written informed consent prior to surgery. Patients have been followed from the time of their operation to the present.

### Surgical Technique

**Fig. 1:**
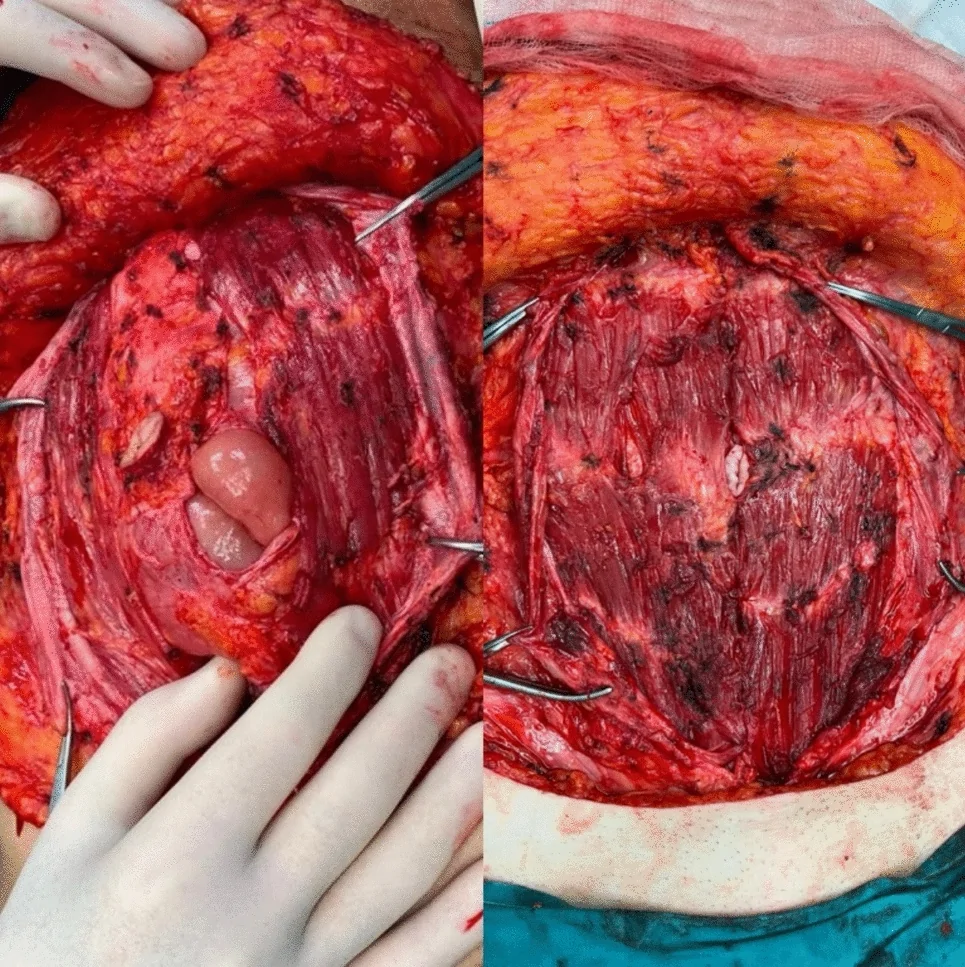
Intraoperative views showing midline hernia before and after rectus muscle approximation. Left: Herniation of intra-abdominal contents through a midline fascial defect with attenuated linea alba and associated diastasis of the rectus abdominis muscles. Right: Primary approximation of the medial rectus bellies using interrupted 2-0 absorbable Vicryl (polyglactin 910) sutures. The anterior wall of the rectus sheath remains open laterally at this stage, pending final fascial closure

**Fig. 2:**
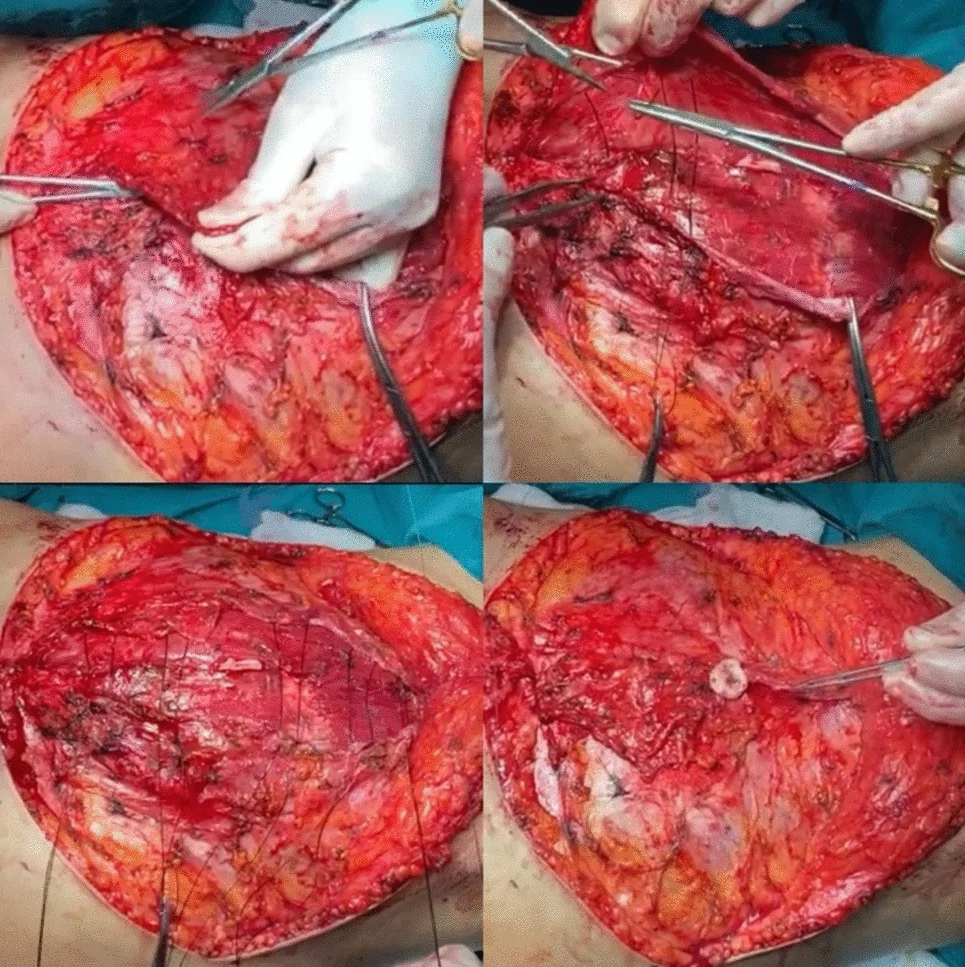
Intraoperative sequence demonstrating overlapping anterior wall of the rectus sheath closure in a modified Mayo repair. (Top left) Lateral dissection of the anterior wall of the rectus sheath enables 5–8 cm of medial advancement on each side. (Top right and bottom left) The anterior rectus sheaths, separated from the underlying rectus muscles, are advanced medially to achieve ≥5 cm overlap and reinforced with a first row of interrupted 1.0 nonabsorbable sutures (placed 4–5 cm from the free edge of the anterior sheath and secured 0.5 cm from the posterior sheath margin). (Bottom right) A second layer of interrupted sutures is placed in parallel to complete the two-layered aponeurotic closure. The umbilical stalk is preserved and reattached at the end of the repair

**Fig. 3:**
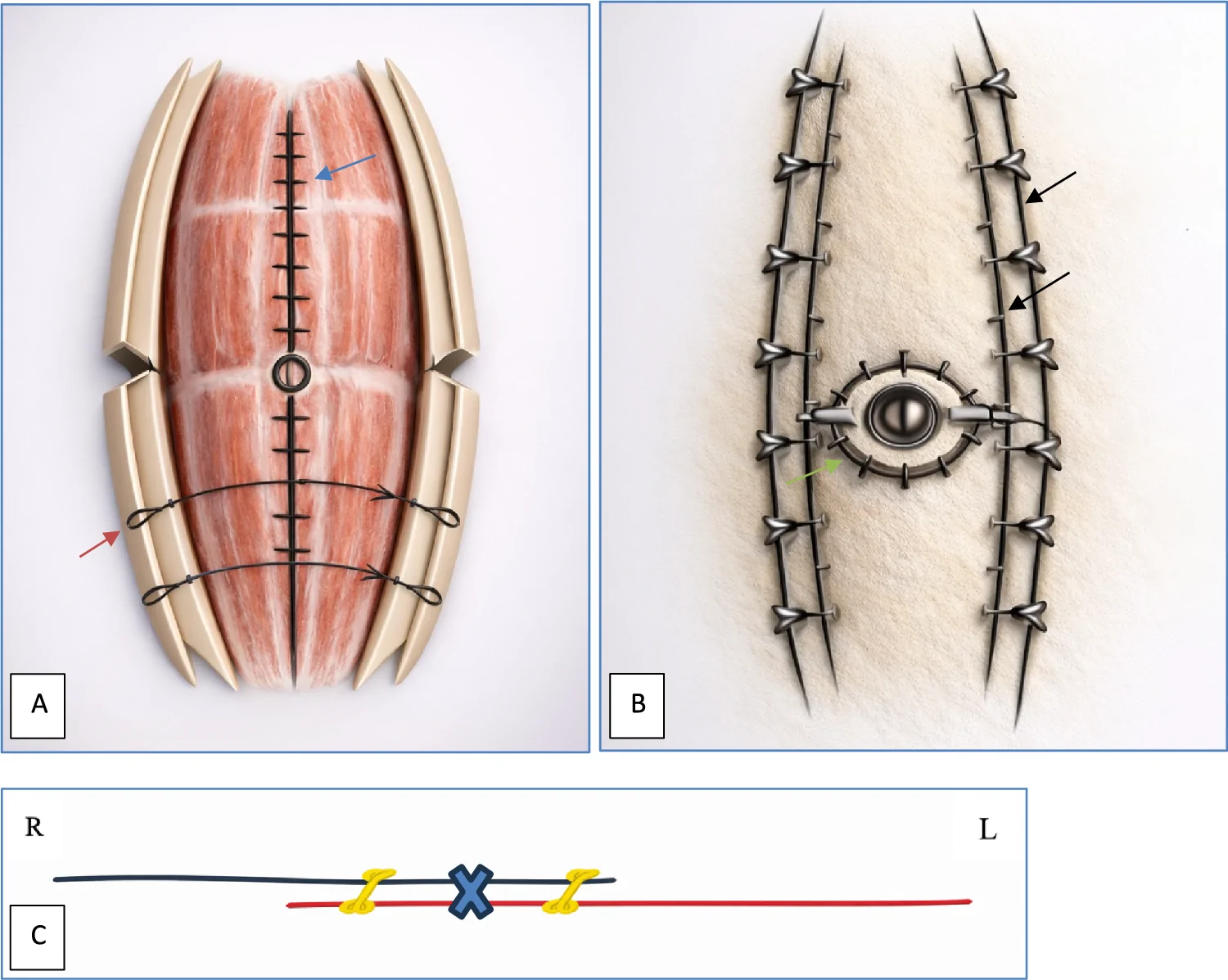
**A**. Midline approximation of the rectus abdominis muscles (blue arrow). The anterior rectus sheath is mobilized and advanced medially on either side. The fascial flaps are overlapped, and interrupted sutures are placed from the free edge of the anterior rectus sheath (red arrow) to the “deep surface” of the contralateral flap, creating a double-layer (modified Mayo) repair around the preserved umbilical stalk. **B**. Final configuration showing the double-layer closure. The suture lines reinforce the midline and stabilize the fascial reconstruction. The umbilical stalk is preserved and secured with interrupted sutures (green arrow). **C**. Schematic representation of a transverse section at the level of the umbilicus showing the double-layer interrupted suture placement on the overlapping flaps of the anterior rectus sheath (with X representing the umbilical stalk)

The patient lies supine on the operating table. The abdominoplasty markings are placed starting from the center of the natural suprapubic crease and are directed laterally toward the anterior superior iliac spines (ASIS) on each side, forming the inferior abdominal incision line. The length of the incision is adjusted according to individual patient anatomy to avoid dog-ear deformities. With the patient flexed at the waist, the superior incision line is marked using the pinch technique, ideally just above the umbilicus. The patient is then returned to the supine position. The initial incision is performed along the inferior marking within the suprapubic crease, extending laterally toward the anterior superior iliac spines. Dissection proceeds through the subcutaneous tissue down to the anterior abdominal fascia. The abdominal flap is then elevated in the plane superficial to the fascia, with cephalad undermining toward the umbilicus and xiphoid process as required, enabling full exposure of the anterior abdominal wall and identification of hernia defects. A rhomboid periumbilical incision is made, and the umbilical stalk is carefully dissected from the surrounding subcutaneous tissue, preserving its attachment to the underlying fascia. Dissection is carried out close to the stalk to maintain the integrity of the deep perforating vessels, which arise from the deep inferior epigastric vessels and constitute the primary vascular supply following flap elevation. The abdominal flap is elevated and resected en bloc. The patient is again flexed slightly at the waist to facilitate tension-free advancement. Further wide undermining is performed cephalad toward the xiphoid process and costal margins to enable adequate flap mobilization. In most cases, the hernia sac is encountered along the line of the midline incision. Adhesions between the bowel and anterior abdominal wall are lysed as needed. Hernia contents are reduced, and the peritoneum is closed using interrupted synthetic non-absorbable 2.0 monofilament sutures. The anterior wall of the rectus sheath is incised along its medial border, exposing the rectus muscles bilaterally. This incision extends from the xiphoid to the pubic symphysis. The anterior wall of the rectus sheath is then dissected laterally as needed to allow 5–8 cm of medial advancement. The rectus muscles are re-approximated in the midline with interrupted 2.0 absorbable Vicryl polyglactin sutures, incorporating the medial edge of the diastasis to restore continuity of the abdominal wall. At this stage, the anterior rectus sheath remains open laterally prior to definitive fascial reconstruction (Fig. 1). The anterior rectus sheaths, separated from their underlying muscles, are then advanced medially to achieve at least 5 cm of overlap. The umbilical stalk is preserved on its vascular pedicle and remains attached to the underlying fascia throughout the procedure, maintaining perfusion from the deep inferior epigastric perforators. Following circumferential periumbilical dissection, the anterior rectus sheath flaps are advanced medially. A small, tailored opening may be created in the advancing fascial flap to accommodate the umbilical stalk without tension. Fascial closure is then completed around the stalk, preserving vascular integrity while achieving secure midline reinforcement. Closure is reinforced using a modified overlapping Mayo repair [14]. Interrupted 1.0 non-absorbable sutures are placed 4–5 cm from the free edge of the anterior sheath and secured to the posterior flap 0.5 cm from its margin. A second reinforcing layer of sutures is placed in parallel, creating a two-layered aponeurotic reconstruction that distributes tension across the repair (Fig. 2). The umbilical stalk is anchored to the aponeuroses in four quadrants: superiorly, inferiorly, and bilaterally, with interrupted sutures. The abdominal cavity is irrigated with saline and checked for hemostasis. With the patient flexed to 45 degrees, the abdominal flap is advanced caudally and approximated to the inferior incision line. A temporary midline 3.0 nylon suture is placed to align the flaps. The new umbilical site is marked using methylene blue and a Pitanguy flap demarcator. A rhomboid incision is created, and the umbilical stalk is exteriorized and inset using partially buried 4.0 non-absorbable monofilament horizontal sutures. One or two multi-perforated drains are positioned beneath the flap, midway between the ASIS and the midline, and secured with 3.0 nylon sutures. Subcutaneous tissue is closed with interrupted 3.0 absorbable Vicryl sutures, and skin closure is achieved using an intradermal running suture or staples. The incision is reinforced with aseptic tape (3M). A schematic representation of the key steps of fascial advancement and overlapping closure is provided (Fig. 3A–C) to enhance clarity of the described technique.

### Postoperative Care

At the end of the procedure, patients were transferred to a hospital bed and positioned with flexion at the waist and elevation of the knees to reduce tension on the abdominal wall. Prophylactic antibiotics were administered, and intravenous fluids replaced as needed. Compression garments were applied immediately and worn continuously for the first two weeks, to reduce seroma formation and support abdominal wall healing. Thromboprophylaxis was provided with TED stockings, and early mobilization was encouraged after the first 24 hours. On postoperative day two, patients were allowed to shower and instructed to change dressings to maintain clean and dry wound sites. Drains were removed once 24-hour output was below 50 mL, typically on postoperative day two. Umbilical sutures were removed between 10 and 15 days postoperatively.

## Results

**Fig. 4:**
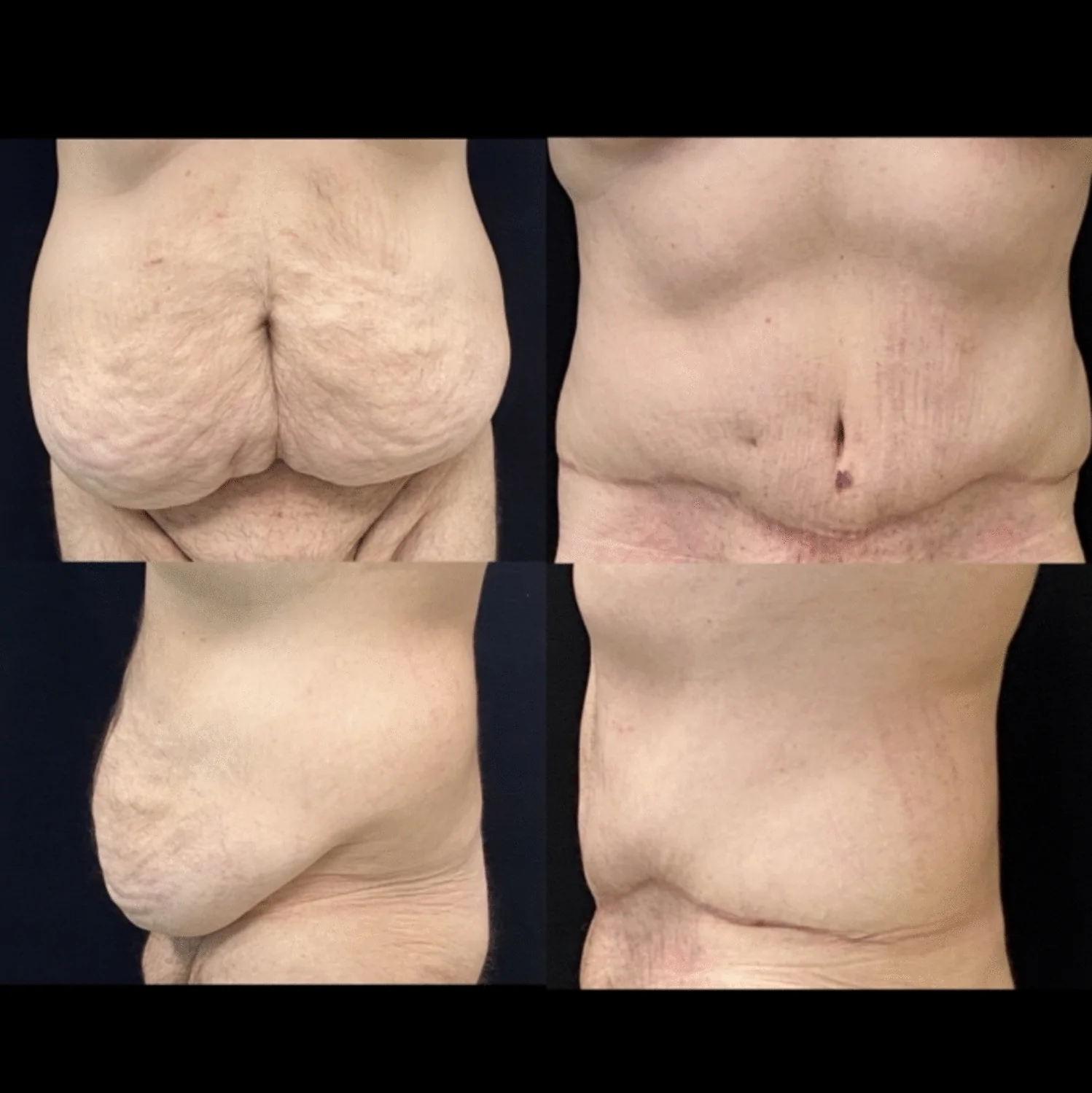
Preoperative and postoperative views of a male patient. Preoperative images (left column) demonstrate severe abdominal wall laxity, significant rectus diastasis, and an infraumbilical midline hernia. Postoperative images (right column) at 6 months follow-up show restoration of abdominal wall contour and musculofascial integrity, achieved via mesh-free reconstruction using a modified Mayo technique. Aesthetic outcomes demonstrate improved skin envelope and tension-free closure

**Fig. 5:**
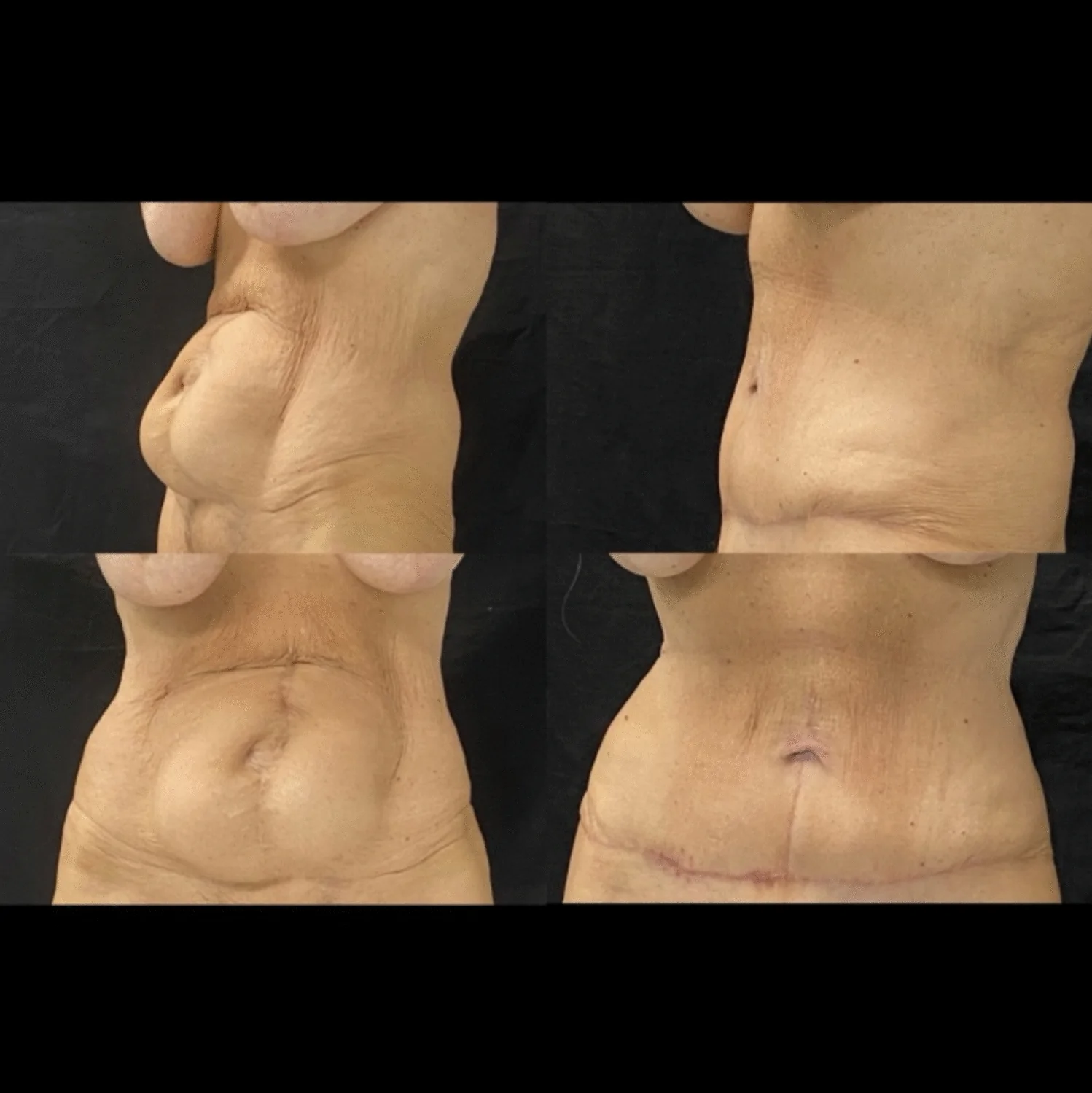
Preoperative and postoperative appearance of a female patient undergoing abdominoplasty with concurrent repair of midline abdominal wall defects. Preoperative photographs (left column) demonstrate severe skin laxity, rectus diastasis, and a prominent paraumbilical hernia. Postoperative images (right column), taken 6 months after surgery, reveal successful anatomic and aesthetic restoration through direct fascial plication and skin resection without mesh use. The umbilicus was preserved and repositioned, and the final scar is low and symmetric

Between 1986 and 2021, a total of 120 abdominoplasties with concomitant repair of diastasis recti and abdominal wall hernias were performed. All patients were discharged within 72 hours postoperatively with standard post-discharge instructions. Postoperative results at 6-month follow-up demonstrated restoration of abdominal wall integrity and improved contour (Figs. 4 and 5). No recurrences of DRAM or hernias were observed during the follow-up period, and no cases of vascular compromise were reported. Follow-up duration ranged from 1 to 39 years, with all patients monitored clinically and photographically. A total of 24 patients (20%) developed postoperative seromas, all of which were successfully managed either by percutaneous drainage or with Penrose drains.

## Discussion

Abdominoplasty is one of the most commonly performed procedures in plastic surgery. Many patients seeking abdominoplasty also present with midline abdominal bulging, often resulting from thinning and widening of the linea alba rather than a true hernia. Their primary concern is typically a deformed abdominal contour caused by a combination of excess lax skin and a weakened musculoaponeurotic system. Diastasis of the rectus abdominis muscles (DRAM) is frequently seen in post-partum women, particularly in those with early pregnancy, large weight gain, multiple pregnancies, or increased abdominal circumference. In overweight or obese men, DRAM may become apparent following weight loss, as the abdominal wall becomes increasingly relaxed. In DRAM, the transversalis fascia remains intact and no fascial defect is present, distinguishing it from true hernias. Although it poses no risk of strangulation, diastasis results in a smooth midline protrusion that becomes prominent with increased intra-abdominal pressure. Patients concerned about their abdominal contour may present for surgical evaluation and correction. In cases of large (> 5 cm) diastasis or markedly attenuated fascia, our proposed surgical technique provides a means of anatomical correction using autologous tissue. This is achieved without prosthetic mesh, instead relying on a reinforced fascial closure via a modified overlapping double-suture Mayo technique. This double-layer repair strengthens the weakened midline and allows the formation of fibrotic tissue for long-term reinforcement. The result is a durable reconstruction of the abdominal wall using local fascia and muscle, improving both aesthetics and core stability while minimizing recurrence. While mesh may reinforce the abdominal wall, it does not address the underlying distention and tissue laxity responsible for the contour deformity.

In some cases, incidental midline hernias were encountered intraoperatively. Traditionally, abdominoplasty and hernia repair are performed in two stages. Combining both procedures offers an anatomic and visual advantage, enabling direct identification and correction of fascial defects while restoring abdominal contour. The technique described here effectively addresses small and medium midline hernias (<4 cm) in conjunction with abdominoplasty.

In our series, all patients were followed up long term, ranging from 1 to 39 years. No recurrences of DRAM or hernia were reported, supporting the effectiveness of this technique in restoring abdominal wall integrity. Although mesh-based repair has been associated with lower recurrence rates in ventral hernia surgery, the patient population in this series differs significantly from those typically included in hernia trials. The majority of defects addressed were small to moderate midline hernias in the setting of diastasis recti, rather than large or complex ventral hernias. Importantly, fascial quality was assessed intraoperatively, and mesh-free repair was performed only in cases where the native tissue was deemed sufficiently robust to support tension-free closure. The technique described allows for wide fascial mobilization and significant overlap (≥ 5 cm), combined with a two-layered imbricated closure, which may enhance biomechanical strength. Additionally, careful patient selection likely contributed to the favorable outcomes, as patients with large, complex, or recurrent hernias or poor fascial integrity were not included. Therefore, the absence of recurrence in this series should be interpreted within the context of patient selection, intraoperative judgment of fascial quality, and surgical technique, rather than as a direct comparison to mesh-based repair in all ventral hernia cases. Seroma formation occurred in 20% of patients, attributed to extensive flap undermining. These were successfully managed with paracentesis and resolved within two weeks. Serosanguineous collections remain the most common complication of abdominoplasty and can be mitigated by the use of compressive garments and drain removal once daily output falls below 50 mL [15].

In patients undergoing concomitant umbilical hernia repair, no vascular compromise or recurrence was observed. The umbilicus is known to have a dual blood supply via the deep inferior epigastric artery and the subdermal plexus [3]. Circumferential skin incisions during abdominoplasty disrupt the superficial supply from the subdermal plexus, leaving the deep perforators as the sole source of vascularization. Preservation of these vessels through meticulous dissection prevents ischemic complications and maintains umbilical viability.

This study has several limitations. It represents a retrospective, single-surgeon experience without a control group. Selection bias is likely present, as only patients deemed suitable for mesh-free repair were included. The absence of standardized imaging follow-up may underestimate subclinical recurrence. Despite these limitations, the extended follow-up and consistency of the surgical technique provide valuable insight into the durability of this approach.

## Conclusion

Abdominoplasty is one of the most sought-after procedures in plastic surgery, particularly for patients with excess abdominal skin and a weakened musculoaponeurotic wall. This patient population frequently presents with diastasis of the rectus abdominis muscles and, in some cases, incidental midline abdominal wall hernias. Preoperative or intraoperative evaluation enables the planning of a combined surgical approach to correct both the diastasis and any hernias in a single-stage procedure. Using the surgical technique described, the abdominal wall is reconstructed with local muscle and fascia through a modified overlapping Mayo repair, addressing both laxity and midline distention without the need for prosthetic mesh. This method resulted in no recurrences of DRAM or hernias, no vascular compromise, and consistently amplified aesthetic outcomes. Our long-term follow-up confirms the durability and safety of this mesh-free approach. High patient satisfaction and absence of major complications further support the viability of this technique as a reliable option for both functional restoration and aesthetic enhancement.
